# A Real-Time Vessel Detection and Tracking System Based on LiDAR

**DOI:** 10.3390/s23229027

**Published:** 2023-11-07

**Authors:** Liangjian Qi, Lei Huang, Yi Zhang, Yue Chen, Jianhua Wang, Xiaoqian Zhang

**Affiliations:** School of Mechanical Engineering, Nanjing Forestry University of China, Nanjing 210037, China; qiliang@njfu.edu.cn (L.Q.); njfujdzhangyi@njfu.edu.cn (Y.Z.); bivid@njfu.edu.cn (Y.C.); 18816212079@163.com (J.W.); zxiaoqian@njfu.edu.cn (X.Z.)

**Keywords:** target tracking, vessel detection, real-time systems, clustering algorithms

## Abstract

Vessel detection and tracking is of utmost importance to river traffic. Efficient detection and tracking technology offer an effective solution to address challenges related to river traffic safety and congestion. Traditional image-based object detection and tracking algorithms encounter issues such as target ID switching, difficulties in feature extraction, reduced robustness due to occlusion, target overlap, and changes in brightness and contrast. To detect and track vessels more accurately, a vessel detection and tracking algorithm based on the LiDAR point cloud was proposed. For vessel detection, statistical filtering algorithms were integrated into the Euclidean clustering algorithm to mitigate the effect of ripples on vessel detection. Our detection accuracy of vessels improved by 3.3% to 8.3% compared to three conventional algorithms. For vessel tracking, L-shape fitting of detected vessels can improve the efficiency of tracking, and a simple and efficient tracking algorithm is presented. By comparing three traditional tracking algorithms, an improvement in multiple object tracking accuracy (MOTA) and a reduction in ID switch times and number of missed detections were achieved. The results demonstrate that LiDAR point cloud-based vessel detection can significantly enhance the accuracy of vessel detection and tracking.

## 1. Introduction

With the advancement of the economy, the usage of waterborne transportation is steadily increasing, making the monitoring of vessels on waterways of paramount importance [1]. However, conventional vision-based detection and tracking algorithms face limitations, particularly in low-light conditions such as adverse weather, fog, and nighttime scenarios. These algorithms typically rely on grayscale or color information within images [2], but the fluctuation in lighting conditions can pose challenges in maintaining the accurate tracking of targets. In regions characterized by dense maritime traffic or congested waterways, visual-based detection and tracking systems may encounter difficulties related to target occlusion and mutual interference, thereby significantly affecting the accuracy of vessel detection and tracking.

In contrast, point cloud data inherently represent objects in three-dimensional space, providing comprehensive spatial information. This becomes invaluable for tasks involving three-dimensional scenes, occlusions, and objects that are partially obscured. Light detection and ranging (LiDAR) technology, which utilizes radio waves for precise distance and positional measurements, enables high-precision vessel detection and tracking. LiDAR is immune to lighting conditions and can operate efficiently even in adverse weather or low-light environments.

Furthermore, the inherent continuity of spatial positions and geometric information within point cloud data allows for the seamless integration of target detection and tracking functionalities. Leveraging point cloud data holds the potential to overcome the limitations of vision-based algorithms in specific environments, thereby enhancing the precision of maritime vessel monitoring. The implementation of LiDAR has the potential to mitigate some obstacles associated with target tracking that are typically encountered in conventional image-based methodologies. Hence, the present study opted to employ the LiDAR point cloud-based detection and tracking approach. 

Deep learning is currently developing very rapidly in target detection [3,4]. Numerous algorithms have been developed to accomplish precise detection in various applications, including vessel, vehicle, and face detection. PointNet [5] has emerged as a pioneering approach for deep learning on point clouds. It is an interactive auto-encoder that incorporates point clouds and images [6] or a center-based detection [7]. PointRCNN [8] improves the target detection performance of PointNet. It introduces techniques such as multi-level feature learning, anchor-based detection strategies, and point cloud segmentation and extraction. To detect 3D objects more accurately with improved speed and stability, PointRCNN specializes in the processing of point cloud data and is typically applied to static 3D point cloud data collected based on LiDAR or other sensors. YOLO3D [9] usually uses a point cloud or depth image as input. It can process data from multiple consecutive frames and is suitable for 3D object detection in dynamic scenes. Many vessel detection and tracking algorithms have also been proposed in recent years [10,11]. 

Wang’s introduction of CFE modules into YoloV3 improved its accuracy [12]. Chen proposed a detection algorithm for small vessels [13]. K.V. Ramachandra used Kalman filtering for tracking [14]. Directly processing LiDAR point clouds was proposed to achieve accurate and stable multi-object tracking [15]. They are independent of each other. Instead, the huge amount of computation required for deep learning does not guarantee that the algorithm can run in real time together with the tracking algorithm, making it difficult to guarantee the real-time performance of the algorithm. Therefore, for this paper, we used some optimized classical algorithms to achieve real-time detection and tracking. Firstly, we clustered the vessels and obtained the bounding box of the vessels by L-shape [16] fitting. After obtaining the bounding box, we calculated the information of the vessels and stored it in our structure for the subsequent calculation. To increase the computational efficiency of the method, we also projected the point cloud and computed part of the information in the projected point cloud [17], which improved computational efficiency. 

In this paper, the related work conducted before performing detection and tracking is presented in Section 2, and the main elements of the algorithm are described in detail in Section 3. The final experimental results are placed in Section 4. Conclusions are drawn in Section 5, and we discuss the limitations of the algorithm.

## 2. Related Work

### 2.1. Point Cloud Cluster Methods

Point cloud clustering plays a significant role in the processing of point clouds. Point cloud clustering serves the purpose of segmenting point cloud data into distinct objects or parts of a scene. Through point cloud clustering, valuable feature information can be extracted from the point cloud data, while noise points and anomalies can be effectively separated from valid points.

The application of Euclidean clustering for point cloud clustering is a well-established and classical approach explored in [18]. Fast Euclidean clustering (FEC) employs a point-wise scheme to enhance the performance of Euclidean clustering [19]. Wen optimized the structure of the Euclidean clustering algorithm and improved its operational efficiency [20]. In [21], researchers introduced a probabilistic framework that integrates both the Euclidean spatial information and the temporal information derived from consecutive frames.

Density-based spatial clustering of applications with noise (DBSCAN) is an unsupervised clustering algorithm first proposed in [22]. In [23], the author proposed a method where the point cloud data are partitioned into grid cells to enhance the scalability and efficiency of the clustering algorithm. The DBSCAN algorithm requires the manual selection of two parameters, which have an important impact on the quality and stability of clustering results. Parameter-adaptive DBSCAN based on density-reachable distance automatically selects the appropriate parameters by estimating the density-reachable distance. The sensitivity of the DBSCAN algorithm to noise points is one of its limitations. Density peaks DBSCAN-based clustering [24] and noise-filtering DBSCAN-based clustering methods introduce more flexible noise point handling mechanisms and improve the robustness of noise points.

Point clouds are disordered points, and it is difficult for us to define objects based on the original point cloud. Inspired by the concept of super-pixels in images, the use of super-voxels in European space is gradually emerging [25], incorporating over-segmentation into super-voxel clustering for better accuracy.

### 2.2. Vessel Tracking 

Simple online and real-time tracking (SORT) [26] was proposed mainly for associating objects efficiently online and in real-time applications. The authors also added the deep association matrix to SORT [27], achieving overall competitive performance at high frame rates. However, SORT uses traditional methods such as Kalman filtering, which may not perform well for situations where the appearance of the target is highly variable, such as target occlusion, illumination changes, etc. The IOU-Tracker algorithm utilizes target association techniques based on the intersection over union (IOU) of target bounding boxes. This approach offers high real-time performance and robustness in tracking targets and therefore may have limitations for non-rectangular targets, such as circular- or irregularly shaped targets. TransTrack is a transformer-based multi-target tracking algorithm [28]. The transformer network can capture long-range dependencies and contextual information between targets, which is conducive to solving problems such as mutual occlusion and changes in motion patterns between targets. Transformer-based tracking algorithms typically require more computational resources and thus may be less suitable for resource-constrained embedded systems or real-time applications.

## 3. Real-Time Canal Monitoring System

### 3.1. Overview of the Proposed System

Image-based vessel recognition and tracking may be hampered by factors such as obscured weather, resulting in erroneous detection. As a result, this study offers a point cloud-based river vessel monitoring system that can effectively address the issue of inaccurate detection due to occlusion and bad weather. In detection, we used Euclidean clustering to cluster the river point cloud to obtain the independent point cloud of the boats precisely and quickly. We also limited the interference of ripples on the size of the ship during navigation by combining statistical filtering with Euclidean clustering. If the original point cloud data are used for tracking, it is easy to encounter issues such as low tracking efficiency and lost tracking. As a result, we merged the L-shape fitting technique with the original tracking approach, which dramatically improved computing efficiency while requiring no experience or parameter tweaking.

**Figure 1:**
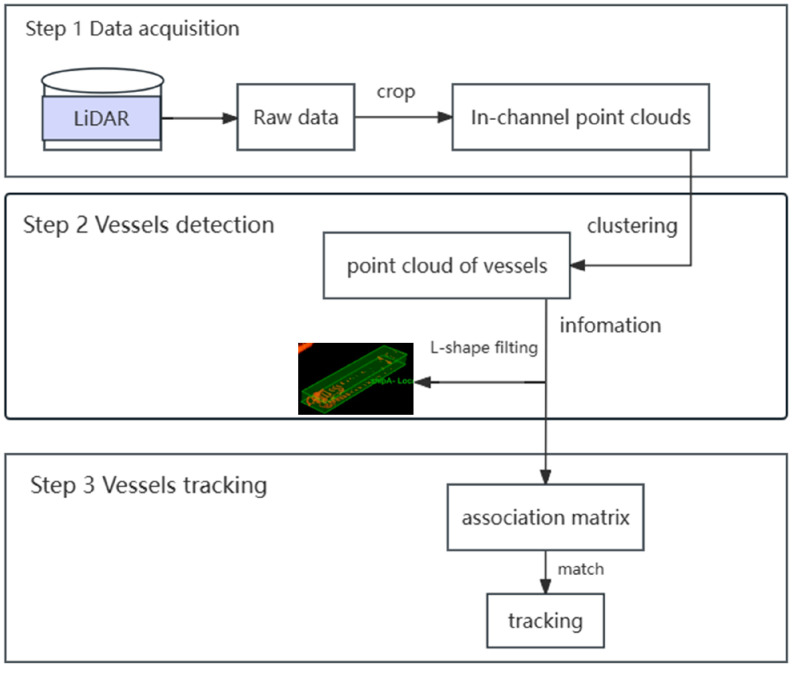
System overview.

In Figure 1, the system structure is depicted.

### 3.2. Vessel Clustering and Fitting

There are many ripples produced by vessels in a channel when there is wind and water. The existence of ripples exerts an influence on later calculations about vessel dimensions. Therefore, filtering the ripples is an essential issue to be solved.

To obtain a better view of the near point of the bridge, we decided to rotate the radar downward by 7° in the *Z*-direction. First, we built the equations for the horizontal plane and provided the starting points. The equation for the plane is as follows:

(1) AX+BY+CZ+D=0

where *A*, *B*, and *C* are the components of the normal vector of the plane and *D* is a constant term of the plane. Then the final parameters *A*, *B*, *C*, and *D* of the horizontal plane are obtained by averaging the plane equations of multiple planes in the past. The horizontal plane is generally oblique, so we can rotate this oblique plane onto the XOY plane. The angle of rotation is:

(2) $$a=\cos^{-1}[\frac{\left(A,B,C)\cdot (0,0,1\right)}{{\left((A,B,C)\cdot (0,0,1)*(0,0,1)\cdot (A,B,C)\right)}^{2}}]$$

where a represents the angle at which the point cloud needs to be rotated. (*A*, *B*, *C*) is the component of the plane normal vector and a is the angle of rotation of the point cloud. *D* is a constant term in the horizontal plane. After extracting the horizontal plane equation of the ripple, the next step is to remove the ripple. This equation traverses all the points in the vessel’s point cloud, puts them into a matrix, and then sorts them to find the farthest and closest points from the horizontal ripple plane. Ripples generally exist at the bottom of the vessel. In our experiments, we verified that ripple removal was most effective when the height was between 20 and 98%. Therefore, we removed the points below 20% to remove the ripples.

To produce unique point clouds for every vessel, the next step is to cluster the vessels to segment the individual point clouds for each vessel. Some frequently used point cloud clustering algorithms are k-means clustering [29], DBSCAN clustering [21], super-voxel clustering [25], Euclidean clustering [30], etc., and a new clustering algorithm is fuzzy clustering [31]. K-means clustering is known for its simplicity and ease of implementation. Additionally, it exhibits high efficiency when handling large data sets. DBSCAN does not require a pre-specified number of clusters, making it highly adaptive to the data. DBSCAN is capable of automatically identifying clusters of arbitrary shapes, regardless of the cluster’s shape. It also handles noisy data efficiently by marking them as outliers. Additionally, DBSCAN excels in handling clusters with varying densities, making it suitable for data sets with clusters of different densities. Super-voxel clustering can handle object details as well as texture and edges, but the algorithm has a high time complexity and the parameter optimization process is more complicated. By comparison, Euclidean clustering performs better in processing high-dimensional data and can effectively handle dimensions. It also has a faster computation speed, which is particularly important. The key to achieving real-time monitoring is to process data quickly. Therefore, Euclidean clustering was very suitable as the clustering algorithm for this system. Moreover, we add statistical filtering algorithms to the Euclidean clustering as in Algorithm 1.
**Algorithm 1:** Optimized Euclidean clustering**Input:** Point Cloud **P**, Radius Threshold **R_th_**, Scaling factor **α** **output:** a list of an index for each point **C** create a kd-tree to present **P**
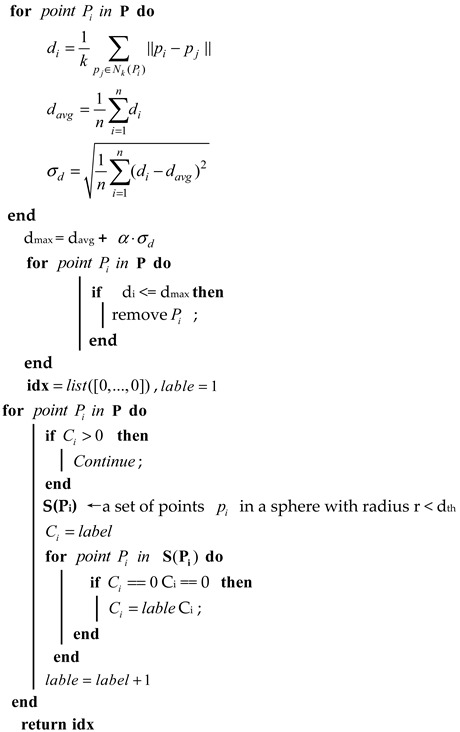


We optimized the Euclidean clustering to be able to remove ripples better during the clustering process. For the input point cloud, we first performed filtering [32] and then clustered it, which can effectively remove the influence of noise.

After clustering, we obtained separate point clouds for each vessel; then, we could calculate their sizes. First, we defined the structure *ShipDetectInfo* that stores the vessel information. The structure stores information about the vessel’s length, width, height, position, and orientation. The parameter *MatchedFlg* was used to determine whether a match was found during tracking. When no match was found, the vessel was added to the list of tracked vessels. We took out the i-th vessel and set it as the input point cloud for projection. We initialized the ripple model coefficients and then projected the vessel to the XOY surface. We rotated the ripple plane to the XOY plane. Next, the information about the vessel was calculated. First, we traversed the unprojected point cloud to find the highest and lowest points of the bridge. The height of the boat equaled the value of the highest point minus the lowest point. The value of the position of the vessel in the z-direction equaled the height of the bridge minus the value of the highest point to the rippling plane.

In the visualization, we needed to show the real-time information of the vessel more clearly, so a 3D bounding box was required. The vessel could be fitted with an L-shape to improve the generation of this 3D bounding box. The principle of L-shape model fitting is to find the rectangle that best fits these points. The specific algorithmic flow we show in Algorithm 2.
**Algorithm 2:** Search-Based Rectangle Fitting**Input:** $\mathrm{range}\;\mathrm{data}\;\mathrm{points}\;X\in R^{n\times 2}$**Output:** $\mathrm{rectangle}\;\mathrm{edges}\;\{a_{i}{x+b}_{i}x=c_{i}|i=1,2,3,4\}$1: Q←02: for θ=0 to π/2-δ step δ do$3:\;{\hat{e}}_{1}\leftarrow (\cos\theta ,\sin\theta )$   ⊳ rectangle edge direction vector$4:\;{\hat{e}}_{2}\leftarrow (-\sin\theta ,\cos\theta )$$5:\;C_{1}\leftarrow X\cdot {\hat{e}}_{1}^{T}$         ⊳ projection onto the edge$6:\;C_{2}\leftarrow X\cdot {\hat{e}}_{2}^{T}$$7:\;q\leftarrow \mathrm{CalculatecriterionX}(C_{1},C_{2})$8:     insert q into Q with key(θ)9: end for$10:\;\mathrm{select}\;\mathrm{key}\;(\theta^{*})$ from Q with maximum value$11:\;C_{1}^{*}\leftarrow X\cdot {\left(\cos\theta^{*},\sin\theta^{*}\right)}^{T},C_{2}^{*}\leftarrow X\cdot {\left(-\sin\theta^{*},\cos\theta^{*}\right)}^{T}$$12:\;a_{2}\leftarrow -\sin\theta^{*},b_{2}\leftarrow \cos\theta^{*},c_{2}\leftarrow \min\{C_{2}^{*}\}$$13:\;a_{2}\leftarrow -\sin\theta^{*},b_{2}\leftarrow \cos\theta^{*},c_{2}\leftarrow \min\{C_{2}^{*}\}$$14:\;a_{3}\leftarrow \cos\theta^{*},b_{3}\leftarrow \sin\theta^{*},c_{3}\leftarrow \max\{C_{1}^{*}\}$$15:\;a_{4}\leftarrow -\sin\theta^{*},b_{4}\leftarrow \cos\theta^{*},c_{4}\leftarrow \max\{C_{2}^{*}\}$

The length and width of the vessel were obtained based on the rectangle output by the algorithm. If a vessel is sailing normally and the direction is parallel to the x-axis direction, then the length and width of the vessel can be calculated very simply.

**Figure 2:**
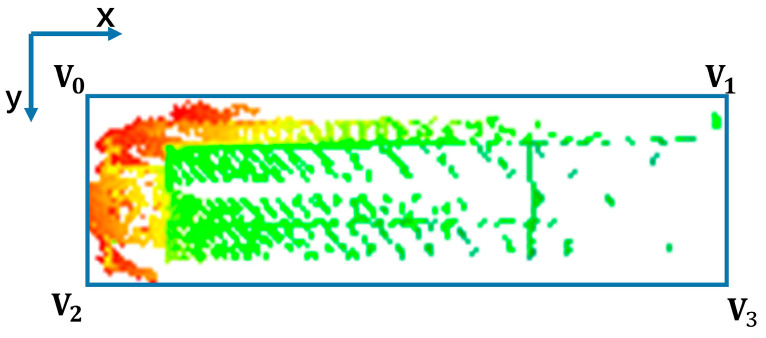
When the vessel is parallel to the x-axis. (The colored point cloud in the blue box is the detected hull point cloud.)

The normal vessel position is shown in Figure 2. We simply iterated through the y and x values of the vertices and then used the maximum value of x minus the minimum value of x to obtain the length of the boat and the maximum value of y minus the minimum value to obtain the width of the boat.

**Figure 3:**
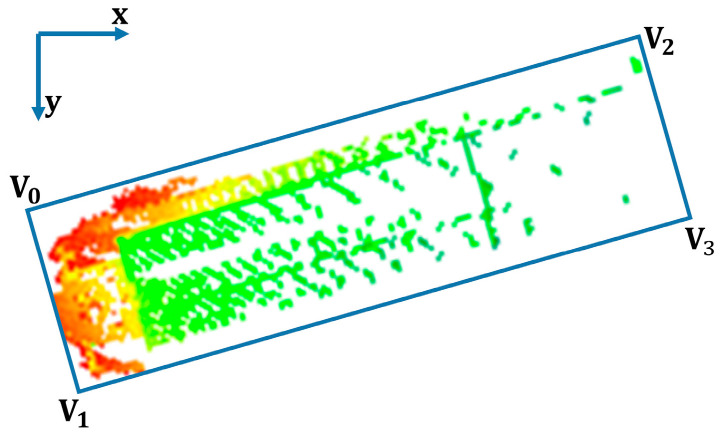
The vessel is not parallel to the x-axis. (The colored point cloud in the blue box is the detected hull point cloud.)

However, in our experiments, we found that some boats were not always parallel to the x-axis, like in Figure 3, so we needed to optimize the calculation of the vessel’s width and length. We then traversed the four vertices according to the value of x. The width of the vessel was the L2 norm of the lineV_0_V_1_. Then, the length, position x, and position y of the vessel were as in Equations (3)–(5):

(3) $$length=\min\{(\sqrt{{\left(x_{v_{0}}-x_{v_{1}}\right)}^{2}+{\left(y_{v_{0}}-y_{v_{1}}\right)}^{2}}),(\sqrt{{\left(x_{v_{0}}-x_{v_{3}}\right)}^{2}+{\left(y_{v_{0}}-y_{v_{3}}\right)}^{2}})\}$$

(4) $$LocX=(x_{v_{0}}+x_{v_{2}})/2$$

(5) $$LocY=(y_{v_{0}}+y_{v_{2}})/2$$

We also calculated the angle of the four sides of the vessel relative to the x direction, where the smallest angle is the declination of the current course. Declination in x-direction is:

(6) $$A_{\min}=\min\{\arctan(\frac{abs(y_{v_{0}}-y_{v_{1}})}{abs(x_{v_{0}}-x_{v_{1}})}),\arctan(\frac{abs(y_{v_{0}}-y_{v_{2}})}{abs(x_{v_{0}}-x_{v_{2}})})\}$$

### 3.3. Vessel Tracking

The traditional vessel tracking system is usually implemented by satellite tracking technology [33,34]. The satellite tracking technology needs to have a GPS receiver on the vessel to receive the signal from the satellite and return the signal to the ground control center [35], which has the advantage of wide coverage but the signal may be interfered with. For this paper, we used a detection-based tracking system by installing LiDAR on the bridge. The target position information provided by the target detection algorithm can mitigate the effects of target appearance changes, scale changes, occlusions, and complex backgrounds on tracking, providing a more stable tracking performance.

To realize real-time vessel tracking, this paper proposes a simple tracking algorithm. The logic of the algorithm is shown below. There are also velocities in the XYZ directions and a *MatchedCnt* to keep track of the number of matched vessels. When the limit is reached, old vessels will be removed to make space for new ones. With callback from the first frame after the power was on, we initialized *VesselTrackInfo* and *VesselHistory* in the first frame. We added some of the detected vessel information in *VesselDetectInfo* to *VesselTrackInfo* and *VesselHistory*. In the second frame, we marked *MatchedFlg* as false. A double level for the loop was used to calculate the matching degree of vessels in *VesselDetectInfo* and *VesselTrackInfo*. We used two variables, *e*_1_ and *e*_2_, to measure the matching degree, where *e*_1_ measures the change in size and *e*_2_ measures the change in position after the vessel’s state is estimated. Then, we put *m*(*e*_1_, *e*_2_) in the association matrix.

(7) $$e_{1}=(w_{d}-w_{t}{)}^{2}+(h_{d}-h_{t}{)}^{2}+(l_{d}-l_{t}{)}^{2}$$

(8) $$e_{2}={\left(lx_{d}-lx_{t}*\Delta t*V_{x}\right)}^{2}/2+{\left(ly_{d}-ly_{t}*\Delta t*V_{y}\right)}^{2}$$

**Figure 4:**
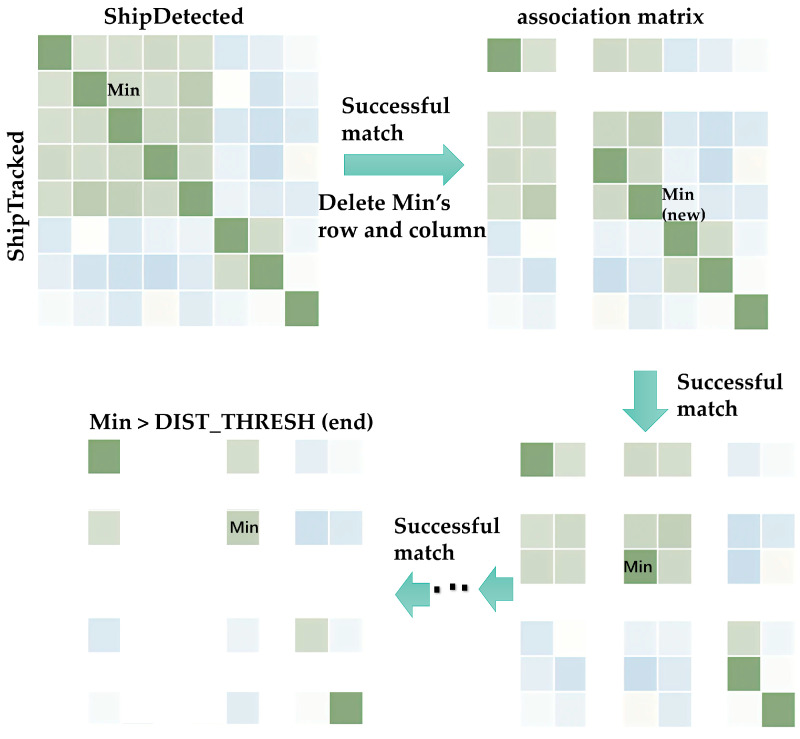
Matching logic of the tracking algorithm.

The rows of the correlation matrix are the numbers of the vessels in *VesselDetectInfo* and the columns are the numbers of the vessels in *VesselTrackInfo*. Through a comparison of the minimum value in the correlation matrix and the correlation threshold, we defined these values. If the minimum value was less than the association threshold, then we marked it as a successful match. We updated *VesselDetectInfo* and *VesselTrackInfo* and then updated the association matrix. We deleted the corresponding rows, as shown in Figure 4, until the minimum value in the matrix was not less than the correlation threshold to jump out of the while loop. If there were vessels in *VesselTrackInfo* that had not been matched, we performed *MatchedCnt*—. Then, we gradually erased the vessel that made out the viewpoint according to the value of *MatchedCnt*. If there was a vessel in *VesselDetectInfo* that was not matched, we added that vessel to *VesselTrackInfo* and *VesselHistory*. If the buffer was full during this process, then we deleted the vessel in *VesselTrackInfo* and *VesselHistory* that had the smallest *MatchedCnt*.

With this tracking algorithm, we can accurately track all boats on a river. We can also plan the path of a vessel if necessary [36].

## 4. Experimental Result

We used a computer configured with an i7-7700 CPU running at 2.8 GHz with 16 GB of RAM and a GTX1050ti GPU card with 4 GB of RAM. The operating system was Windosws10, and the LiDAR was two RS-LiDAR-M1s.

### 4.1. Data Set Acquisition

The vessel data set utilized in this study was obtained from the Lingqu Canal, employing RoboSense’s self-developed, second-generation, intelligent, solid-state LiDAR: RS-LiDAR-M1. The LiDAR device had a horizontal viewing range that extended from −60.0° to +60.0° and a vertical field of view that covered from −12.5° to 12.5°. To optimize the utilization of the LiDAR, a recommended adjustment involved turning it by 7° downwards along the y-axis. This adjustment was expected to result in an expanded field of vision. The PCAP files, which were acquired using the RSView_v3.2.7 (software for viewing and analyzing remote sensing data) recording tool, were then segmented into frame-based pad data by utilizing a callback function that references the LiDAR timestamp.

**Figure 5:**
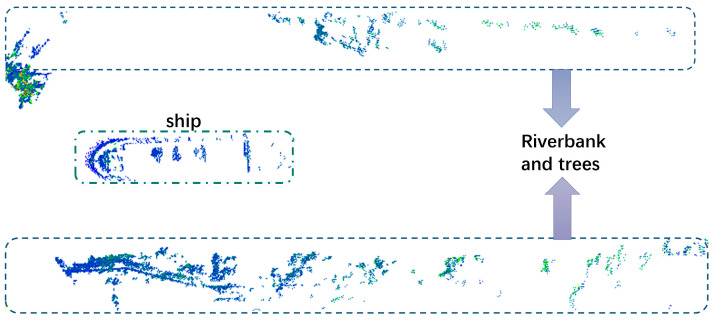
One frame point cloud. (The dotted boxes above and below are riverbanks, and in the middle is the river.)

Figure 5 shows the original point cloud data of a certain frame; we can see that there were many point clouds of trees and riverbanks along the river. This resulted in a considerable volume of data being processed with reduced efficiency. Therefore, we preprocessed the raw data and cropped out the point cloud of the shore.

**Figure 6:**
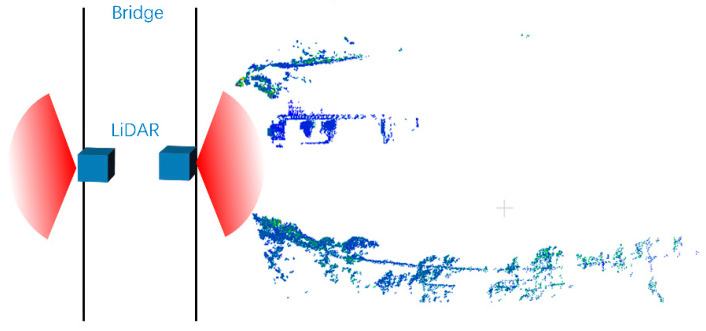
Location of LiDAR and river (two blue boxes are LiDAR devices, and the angle of the red sector is the scanning angle of the LiDAR).

In Figure 6, the bridge is nearly perpendicular to the river channel. We placed one LiDAR device on both sides of the bridge. The point cloud on the right side of the bridge is one frame from the data acquired by the LiDAR.

### 4.2. Vessel Detection

We used LiDAR to record data from passing vessels on the river on both sides of the bridge and converted the PCAP (packet capture) file to a PCD (point cloud data) file for each frame. We obtained about 50 PCAP files with about 24,000 frames of PCD data for the experiment.

**Figure 7:**
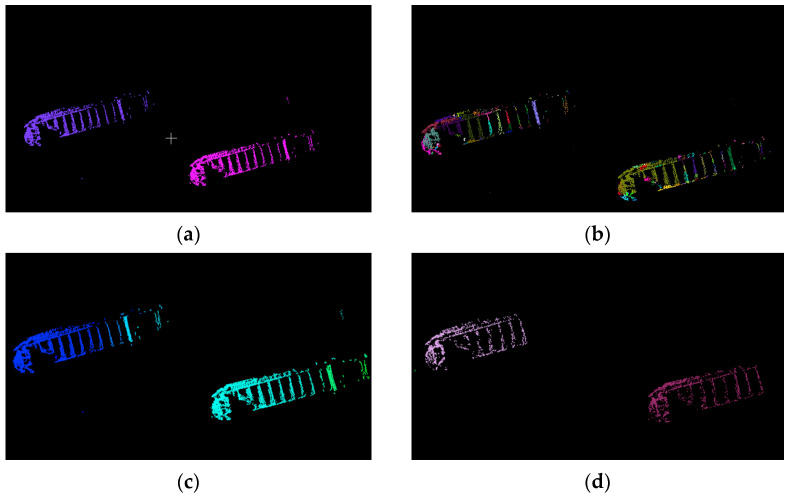
The effect diagram of four clustering algorithms. Different colors represent different clusters. (**a**) K-means cluster; (**b**) super-voxel cluster; (**c**) DBSCAN; (**d**) Euclidean cluster.

The outlines of the riverbanks on both sides of the bridge were different; therefore, both sides needed to be selected separately. According to the above, the proposed Euclidean clustering was more suitable for this experiment than k-means clustering, DBSCAN clustering, and super-voxel clustering. We verified this subsequently, and the clustering results are shown in Figure 7.

As can be seen in Figure 7, k-means and Euclidean clustering had the best performance, clustering two vessels into two clusters. DBSCAN had the following performance: four clusters were clustered. Super-voxel had the worst performance, clustering out more than 10 clusters. However, if we know the number of vessels in advance and give the k-means the exact parameters, the k-means can cluster very well. Most of the time the number of vessels is unknown, so Euclidean clustering is still better than k-means clustering. Processing speed is also an important factor to consider. Euclidean clustering is the structure of a KD-tree with time complexity *Nlog*(*N*). The time complexity of k-means clustering is *kNi*, where k is the number of clusters, N is the number of samples, and the number of iterations is *i*. The time complexity of DBSCAN clustering is *Nlog*(*N*), where N is the number of samples. The time complexity of super-voxel clustering depends on the implementation and parameter settings; we gave parameters of 0.5 m voxel resolution and 8.0 m seed resolution but, in general, the time complexity varies from *Nlog*(*N*) to *N^2^*. Their efficiencies are shown in Table 1.

In Table 1, we can see that the fastest of the four clustering algorithms was the Euclidean clustering; the processing speed per frame of the DBSCAN clustering was only 1.6 ms slower than that of the Euclidean clustering, and the super-voxel clustering performed worse. To attain real-time detection and tracking, it's essential to ensure high-speed performance [37].

Combining the clustering effect and processing speed, we chose Euclidean clustering. For clustering to obtain the hull, we needed to calculate the 3D bounding box of the vessel. We transferred the captain’s width and height to the parameters of the bounding box; then, we could obtain the 3D bounding box of the vessel.

**Figure 8:**
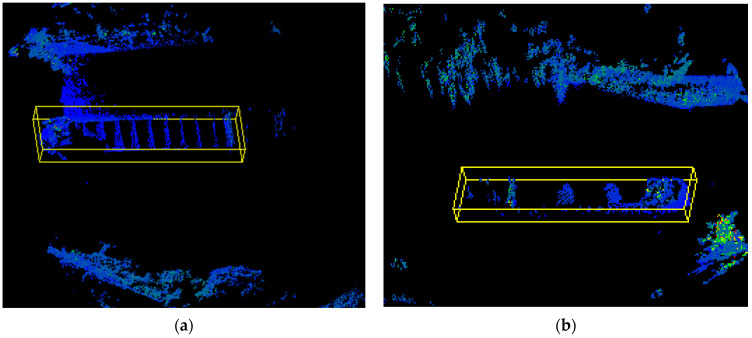
Detection effect on both sides of the river: (**a**) right side; (**b**) left side.

**Figure 9:**
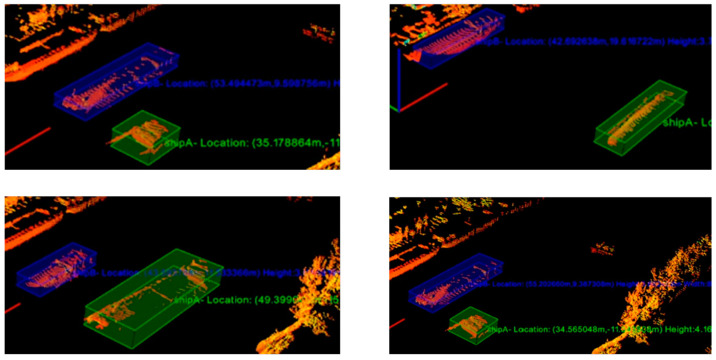
Detection effects of multiple vessels.

As shown in Figure 8 and Figure 9, the 3D bounding boxes were the identified vessel. When multiple ships were present, they were denoted by distinctly colored 3D bounding boxes. As Figure 9 illustrates, it was evident that the system consistently maintained precise detection, even in scenarios involving multiple vessels. To validate the accuracy of our detection algorithm, we compared it with three other algorithms: PointNet [5], YOLO3D [9], and PointRCNN [8]. For each algorithm, we tallied the number of correctly recognized targets as well as the number of false recognitions. The accuracy rate was then computed by dividing the number of correct recognitions by the total number of targets within the field of view, as illustrated in Table 2.

Table 2 indicates that our method demonstrated the lowest number of false identifications and the highest number of correct identifications among the four algorithms. Additionally, our method achieved a higher accuracy rate compared to the other three algorithms.

### 4.3. Vessel Tracking

When tracking vessels, we found that, due to the characteristics of the LiDAR itself, the number of points gradually increased for vessels from far to near and decreased when the vessel was far from sight. This led to the possibility that the size of the vessel could change with the distance. To reduce this error, we added a smoothing algorithm to the processing of the width to remove the noise and make the width closer to the real width.

**Figure 10:**
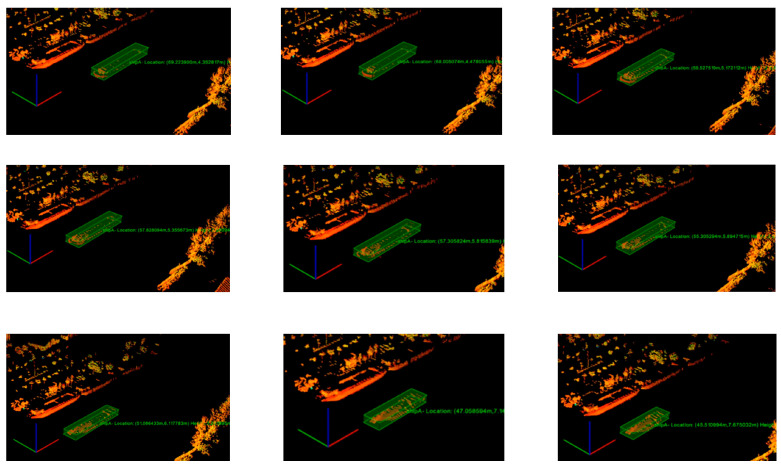
Vessel tracking effects (the order in which the vessels appear in the figure is left to right, top to bottom).

From Figure 10, we can see that the tracking effect of the vessel was more stable during the voyage.

**Figure 11:**
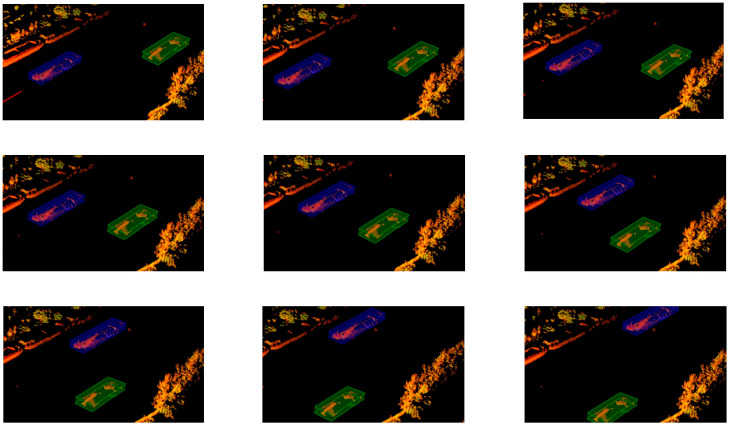
Tracking with multiple vessels.

From Figure 11, it is evident that the vessel IDs remained consistent even in the presence of multiple targets, as indicated by the unchanged color of the enclosing boxes. This observation underscores the system’s strong tracking capabilities.

**Figure 12:**
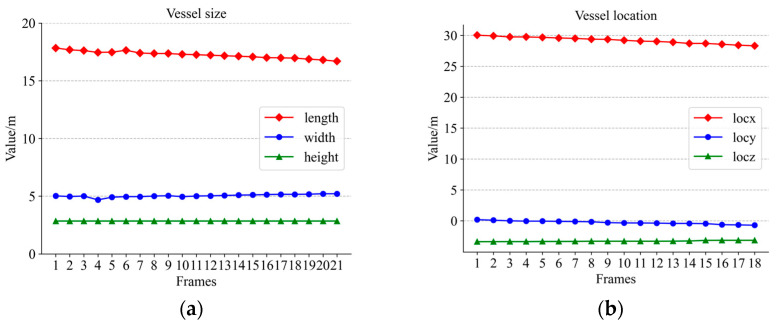
Large-sized vessel information: (**a**) size variation; (**b**) location variation.

**Figure 13:**
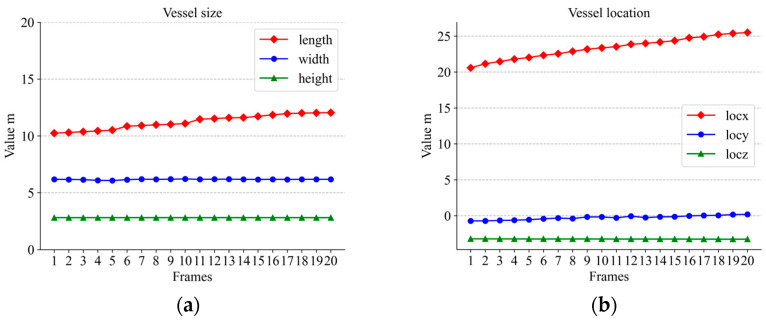
Medium-sized vessel information: **(a)** size variation; (**b**) location variation.

**Figure 14:**
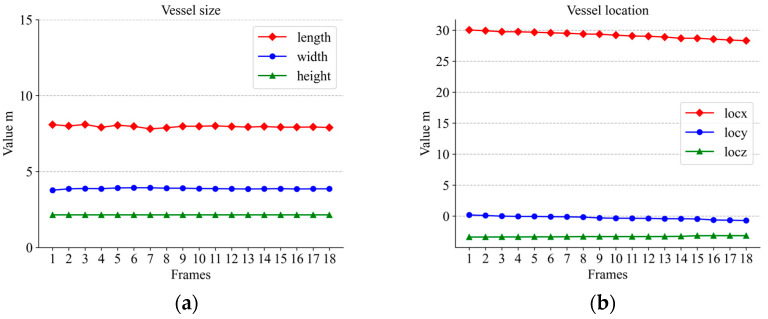
Small-sized vessel information: (**a**) size variation; (**b**) location variation.

Figure 12, Figure 13 and Figure 14 show the variations in the aspect of several vessels within 20 frames. With less fluctuation in size, the size of the calculation was still relatively good. However, there were still some small fluctuations in the data; this is probably because the wind and waves were too strong. Some ripples were not completely removed. But these are acceptable. If the shortest distance between the bounding boxes of two vessels in the field of view is less than the threshold we set and the speed on the vertical bisector of the border is greater than the threshold, the device on the bridge will warn the vessel. In the experiment, the vessel’s ID did not change whenever the vessel was detected and stayed in the middle of the vessel leaving the view, so the tracking effect was very good.

By comparing the detected hull dimensions with the true hull dimensions in Figure 12a, it can be concluded that the width fluctuation of large-sized vessels was controlled to be less than 6.39% of the true value and the length fluctuation was controlled to be less than 3.45% of the true value. Similarly, the comparison between the dimensions in Figure 13a and their true values reveals that the fluctuation of the width for medium-sized vessels was below 13.6% of the true value and the fluctuation of the length was controlled below 2.3% of the true value. Among the three boat sizes, smaller-sized boats exhibited the least fluctuation, with length varying at less than 4.8% of the true value and width fluctuating at less than 1% of the true value, as in Figure 14a. The position change curves of the vessels in Figure 12b, Figure 13b and Figure 14b show that the tracking effect was stable for large-sized vessels.

To validate the performance of the proposed algorithm in multi-object tracking of vessels, we conducted tests on our data set and compared the experimental results with those of the SORT [26], DeepSORT [27], and EAMTT [38] algorithms. The results are presented in Table 3. Due to the complexity of evaluating the multi-target tracking performance using a single score, we employed the evaluation metrics defined in [39] along with the standard metrics used in multiple object tracking (MOT) [40].

Table 3, multi-object tracking accuracy (MOTA), gives a summary of the overall tracking accuracy in terms of false positives, false negatives, and identity switches. Multi-object tracking precision (MOTP) is a summary of the overall tracking precision in terms of the bounding box overlap between ground truth and reported location. Identity switches (IDsw) are the number of times the reported identity of the ground-truth track changed. Table 3 demonstrates the favorable results of our algorithm, particularly in terms of having a low number of target ID switches occurring only once. The MOTA was 1.4% to 10.1% higher than the other three algorithms. MOTP was only 0.1% lower than SORT. Furthermore, our algorithm surpassed two out of the three algorithms in terms of false positives (FP). Our algorithm achieved the best performance in terms of false negatives (FN) among the compared algorithms. Overall, our algorithm demonstrated a consistently stable performance in object tracking.

## 5. Conclusions

This paper proposes a point cloud-based vessel detection and tracking algorithm. The statistical filtering technique was integrated into the Euclidean clustering to effectively address the impact of ripples on vessel detection in the experiment, leading to a significant improvement in the accuracy of vessel detection. Compared with the three traditional algorithms, the vessel detection accuracy was enhanced by 3.3% to 8.3%. Simultaneously, the L-shape fitting of the detected vessels enabled a more accurate acquisition of the size and position information, thus providing a solid foundation for vessel tracking. In terms of tracking, we proposed a simple and efficient algorithm that effectively addressed the ID-switching problem encountered by image-based tracking algorithms during occlusion. Our method demonstrated an accuracy improvement of 1.4–10.1% when compared with three traditional tracking algorithms. Experimental data also showed a reduction in the number of ID-switching occurrences and a decrease in the false detection rate.

Limitation and future work. Due to the nature of LiDAR, as the distance from the LiDAR became farther and farther, fewer and fewer points were obtained, so the long-distance vessels could not be detected. Later, if we want to achieve longer-range vessel tracking, we need to replace the long-range LiDAR with another system such as FMC (frequency-modulated continuous wave) LiDAR. Another limitation is that the ripples could not be removed clearly when the wind and waves were strong, so we need to perform optimization in the clustering algorithm to achieve a more accurate measurement of the vessel’s data even when there are ripples. Alternatively, we may try employing deep learning techniques to detect and remove ripples. The visualization function can be visualized by third-party software provided by LiDAR vendors in combination with our detected vessels. Alternatively, we could use QT (a cross-platform application development framework) programming to better display the current status of the full river channel. This would help to optimize vessel scheduling, improve transport efficiency and safety, and identify abnormal behavior or potential safety risks.

## Figures and Tables

**Table 1:** Inference time of one LiDAR frame.

Methods	Speed of Single Frame
K-means Cluster	25.4 ms
DBSCAN Cluster	17.8 ms
Euclidean Cluster	16.2 ms
Super-voxel Cluster	62.6 ms

**Table 2:** Recognition of the four algorithms in the three scenarios.

Method	Number of Correct Identifications	False Positive	False Negative	Accuracy%
PointNet	493	3	24	81.5%
YOLO3D	502	4	41	80.6%
PointRCNN	511	2	17	85.6%
Ours	523	2	9	88.9%

**Table 3:** Comparison of multi-object tracking effects of different algorithms.

Method	MOTA%	MOTP%	IDsw	FP	FN
EMATT	53.2	77.5	5	13	261
SORT	59.5	79.2	6	26	150
DeepSORT	61.9	78.9	3	29	146
Ours	63.3	79.1	1	18	141

## Data Availability

The data presented in this study are available on request from the corresponding author. The data are not publicly available due to confidentiality agreement.
